# Surgical indication and strategy for liver hemangioma in the caudate lobe: a multi-institutional retrospective analysis with 137 patients

**DOI:** 10.1186/s12957-020-01901-z

**Published:** 2020-06-10

**Authors:** Wei Long Cai, Xiao Ming Ma, Xu Heng Sun, Tai Ren, Cong Yun Huang, Yong Sheng Li, Xu An Wang, Ying Bin Liu, Shu You Peng

**Affiliations:** 1 Huzhou Central Hospital, Affiliated Central Hospital of Huzhou University, 198 Hongqi Road, Huzhou, 313003 Zhejiang Province People’s Republic of China; 2 Department of General Surgery, The second affiliated hospital of Soochow University, Suzhou, 215000 Jiangsu Province People’s Republic of China; 3 Department of General Surgery, Xinhua Hospital Affiliated to Shanghai Jiaotong University School of Medicine, No.1665 Kongjiang Road, Shanghai, 200092 People’s Republic of China; 4 Department of General Surgery, Yuebei People’s Hospital Affiliated to Shantou University School of Medicine, Shaoguan, 512025 Guangdong Province, People’s Republic of China; 5 Department of Hepatobiliary and Pancreatic Surgery, The Second Affiliated Hospital of Zhejiang University School of Medicine, Hangzhou, 310006 Zhejiang Province People’s Republic of China

**Keywords:** Caudate lobe, Liver hemangioma, Indication, Surgical approach, Caudate lobe hemangioma

## Abstract

**Objective:**

To investigate the surgical indication and tactics for liver hemangioma in the caudate lobe

**Methods:**

From January 1994 to July 2019, 137 patients, including 51 males and 86 females with the average age of 49.2 years old were diagnosed with liver hemangioma in caudate lobe and received treatment at five tertiary referral hospitals. Clinical features, correlations between tumor size and clinical manifestations, treatments, and prognosis were analyzed.

**Results:**

Of the 137 patients identified, 40 (29.20%) patients were asymptomatic, whereas other 94 patients had clinical symptoms mainly presented as upper abdominal discomfort, epigastric distention, upper abdominal dull pain, nausea, and vomiting. Fifteen (93.75%), 18 (39.13%), and 7 (10.45%) patients presented no clinical symptoms among those tumor size was less than 3 cm (D ≤ 3 cm, *n* = 16), 3 cm < D ≤ 6 cm (*n* = 46), and 6 cm < D ≤ 9 cm (*n* = 67), respectively, while all 8 patients with tumor larger than 9 cm were symptomatic. Tumor diameter was obviously associated with the presence of clinical symptoms. In follow-up period, 7 patients in the conservative group (*n* = 39) received surgery because of tumor growth or symptom appearance. Totally 105 patients received operation including partial resection or isolated complete resection of caudate lobe and caudate lobe resection combined with liver segment resection, right liver resection, or left liver resection. All operations went smoothly, and no severe complications appeared.

**Conclusion:**

Tumor diameter was obviously associated with the presence of clinical symptoms in patients with hemangioma in caudate lobe. Surgical therapy is not recommended for asymptomatic patients and available for patient who has symptoms. Effective surgical strategies should be put into use to reduce operative bleeding.

## Introduction

The caudate lobe, situated deeply in the liver, between the hepatic hila and inferior vena cava (IVC), is anatomically special which had made it hardly feasible to operate on it. Nowadays, caudate lobe surgery remains a challenge to hepatobiliary surgery and has become a representation of technically demanding hepatobiliary surgeries. As anatomy and medical imaging technology develops, with cutting-edge surgical instruments and liver blood flow control techniques, caudate lobe surgery is not hesitating any more for highly experienced surgeons.

According to reports domestic and overseas, in the meantime, isolated or combined caudate lobectomy is mainly the treatment of liver hemangioma, hepatocellular carcinoma (HCC), metastasis liver carcinoma, hilar hepatic cholangiocarcinoma (HCCA), hepatolithiasis in caudate lobe, etc. Among all those hepatobiliary diseases, liver hemangioma in caudate lobe accounts for a large proportion [1]. However, little common views have been reached about the standard of surgical indication, risk assessment, and selection of treatments for liver hemangioma to the best of our knowledge [2, 3]. Needless to mention that there are rare common opinions about live hemangioma in caudate lobe. Therefore, we collected and reviewed case files of patients diagnosed with liver hemangioma of caudate lobe and treated from January 1994 to July 2019 at five medical centers in China and studied the surgical indication and strategy for liver hemangioma of caudate lobe by analyzing clinical characteristics of the patients.

In this study, we tried to promote the standardization and normalization of therapeutic algorithm for liver hemangioma in caudate lobe and if possible, we hope our study may provoke new ideas and contribute to prevention of overtreatment.

## Patients and methods

### Patient and variable selection

A multi-institutional database of 137 patients treated at five large medical institutions was queried for patients who were diagnosed with liver hemangioma of caudate lobe by imaging examination between January 1994 and July 2019. We summarized and analyzed the information of age, sex gender, clinical manifestation, results of imaging and laboratory examination, location and size of the tumor, treatments, surgical approaches, pathologic results, postoperative complications, etc.

### Treatments

#### Non-operative treatment

Conservative treatment and follow-up in outpatient department is recommended to patients who presented no symptoms or those whose tumor is no more than 3 cm in diameter.

#### Surgical treatment

Operations were performed in patients with clinical features including upper abdominal discomfort, nausea and vomiting, epigastric distention, upper abdominal pain and abdominal mass, patients with capillary angioma-thrombocytopenia syndrome, females preparing for pregnancy, and patients that could not be excluded from malignant disease by imaging examination. Surgical approaches of partial resection for caudate lobe, isolated complete resection for caudate lobe, and caudate lobe resection combined with liver segment resection, right liver resection, or left liver resection were included in our study.

#### Surgical procedures

Of the 105 cases underwent surgery in the study, traditional surgical approaches were taken mainly by the year of 2010 [4, 5], and then laparoscopic caudate lobe resection and the approach guided by four surfaces of caudate lobe were gradually adopted after 2010 [6, 7].

1. Traditional surgical approaches
  Left side approach, or right side approach, or bilateral approach, and anterior transhepatic approach splitting the liver through the middle liver fissure were adopted in the operation as traditional approaches. Operation approaches in detail were recorded in reference [8].
2. The approach guided by four surfaces of caudate lobe
  The four surfaces of caudate lobe are triple portal system in first porta hepatis, division of the top of caudate lobe and middle and left liver vein, short hepatic vein system in third porta hepatis, and conjunctions between caudate lobe and left and right liver [9]. Specific operation techniques could be found in reference [7, 10, 11]. Once the caudate lobe could not be separated from the first three surfaces, anterior transhepatic approach should be considered.
3. Blood flow control maneuvers including preplacing tapes around inferior hepatic IVC and first and second porta hepatis should be adopted as a safety precaution when encountering superior difficulty of operation.
4. Laparoscopic surgical approach is processed with methods above.

### Postoperative care and follow-up

Postoperatively, all patients underwent regular periodic follow-up and reexamination of clinical features, liver and kidney functions, abdominal CT imaging, type B ultrasound etc.

### Ethics approval and consent to participate section

Informed consent was obtained from all participating patients, and the ethics committee of the Xinhua Hospital Affiliated to Shanghai Jiaotong University School of Medicine, Huzhou Central Hospital, the second affiliated hospital of Soochow University, Yuebei People’s Hospital, and the Second Affiliated Hospital of Zhejiang University School of Medicine approved this study.

## Results

### Demographics (Table 1)

In this study, totally 137 patients were treated in five large clinical centers in 25 years. There are similar numbers of male (*n* = 73) and female (*n* = 67) patients with a gender ratio of 1.14:1, whose median age was 49.2 years.

**Table 1:** Clinical characteristics of 137 patients

	*n* = 137	
Age (years)	49.2 (32.5–79.7)	
Gender		
Male	73/137 (53.3%)	
Female	64/137 (46.7%)	
Tumor location		
Spigelian lobe	21/137 (15.3%)	
Para-IVC	10/137 (7.3%)	
Caudate process	12/137 (8.8%)	
Whole caudate lobe	75/137 (54.7%)	
Caudate lobe and left liver	3/137 (2.2%)	
Caudate lobe and right liver	5/137 (3.6%)	
Caudate lobe and liver segments	11/137 (8.0%)	
Tumor size (diameter, D)		Symptom rate
D ≤ 3 cm	16/137 (11.7%)	6.25% (1/16)
3 cm < D ≤ 6 cm	46/137 (33.6%)	60.9% (28/46)
6 cm < D ≤ 9 cm	67/137 (48.9%)	89.6% (60/67)
D > 9 cm	8/137 (5.8%)	100% (8/8)

### Clinical features (Table 1)

Asymptomatic patients (*n* = 40) were discovered accidentally mainly in routine medical examination or in examinations for other disease, whose diagnosis of liver hemangioma in caudate lobe was determined by enhanced CT scanning. The other 97 patients presented clinical symptoms including the most common upper abdominal discomfort (*n* = 35, 36.1%), epigastric distention (*n* = 29, 29.9%), upper abdominal dull pain (*n* = 23, 23.7%), and nausea and vomiting (*n* = 10, 10.3%). Symptomatic patients appear mostly in those whose tumor size is longer than 3 cm in diameter(3 cm < D ≤ 6 cm, 60.9%; 6 cm < D ≤ 9 cm, 89.6%; D > 9 cm, 100%) and only one of eight patients whose tumor size in diameter is no longer than 3 cm presents clinical symptoms.

### Tumor characteristics (Table 1)

Tumor size in diameter (D) is no larger than 3 cm in 16 patients (16/137, 11.7%) of which only 1 patient (1/16, 6.25%) presented right upper abdominal discomfort, 3 cm < D ≤ 6 cm in 46 patients (46/137, 33.6%) of which 28 cases (28/46, 60.9%) have clinical symptoms, 6 cm < D ≤ 9 cm in 67 patients (67/137, 48.9%) of which 60 patients (60/67, 89.6%) presented gastric symptoms, mainly pressure related symptoms, in different levels, and D > 9 cm in 8 patients (8/137, 5.8%) that all presented gastric symptoms mainly as pressure related symptoms companied by pain.

### Treatments (Table 2)

**Fig. 1:**
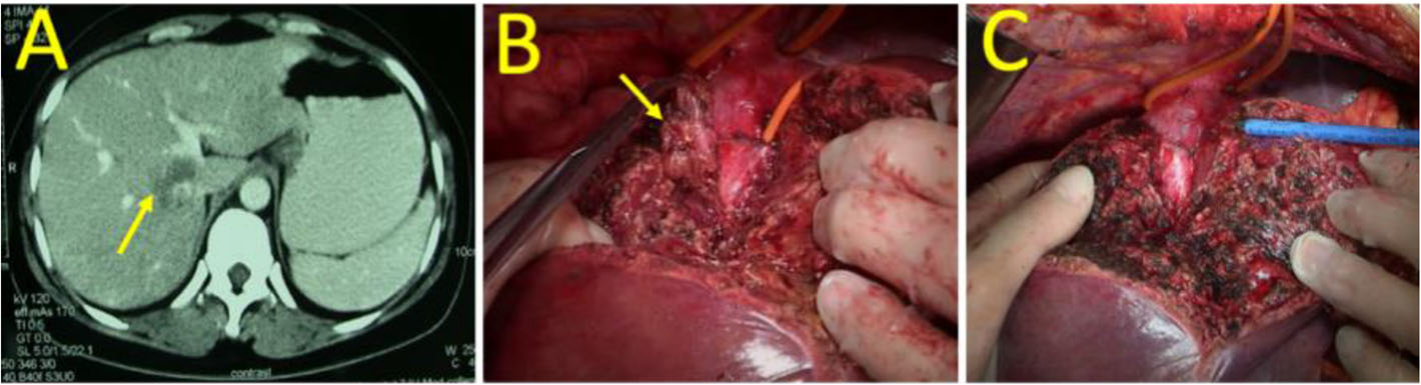
Partial resection of caudate lobe through anterior transhepatic approach (**a** Hemangioma in the caudate lobe aside the IVC. **b** Partial resection of caudate lobe. **c** Hemangioma removed)

There are 39 of 137 patients received conservative treatment and 7 of them were reoperated due to tumor growth and recurrence of clinical symptoms during follow-up. The other 105 cases received surgical treatment. There are 2 patients in D ≤ 3 cm group received operation because of symptoms occurrence in 1 case and possibility of malignant tumor that could not be excluded by imaging examination in the other. Surgical approaches adopted mainly were caudate lobe partial resection, 1 of them received anterior transhepatic caudate lobe resection cutting through the middle liver fissure because of the tumor’s location aside vena cava (Fig. 1).

**Table 2:** Treatments of hemangioma in the caudate lobe

	*n* = 137
Conservative treatment	32/137 (23.4%)
D ≤ 3 cm	14/16 (87.5%)
3 cm < D ≤ 6 cm	16/46 (34.8%)
6 cm < D ≤ 9 cm	2/67 (3.0%)
D > 9 cm	0/8 (0%)
Surgical operation	105/137 (76.6%)
D ≤ 3 cm	2/16 (12.5%)
3 cm < D ≤ 6 cm	30/46 (65.2%)
6 cm < D ≤ 9 cm	65/67 (97.0%)
D > 9 cm	8/8 (100%)
Surgical methods	
Isolated partial caudate lobe resection (anterior transhepatic approach 6 cases)	15/105 (14.3%)
Isolated completed caudate lobe resection (anterior transhepatic approach 29 cases)	71/105 (67.6%)
Combined isolated completed caudate lobe resection	19/105 (18.1%)
Combined with left liver	3
Combined with right liver	5
Combines with liver segments	11

**Fig. 2:**
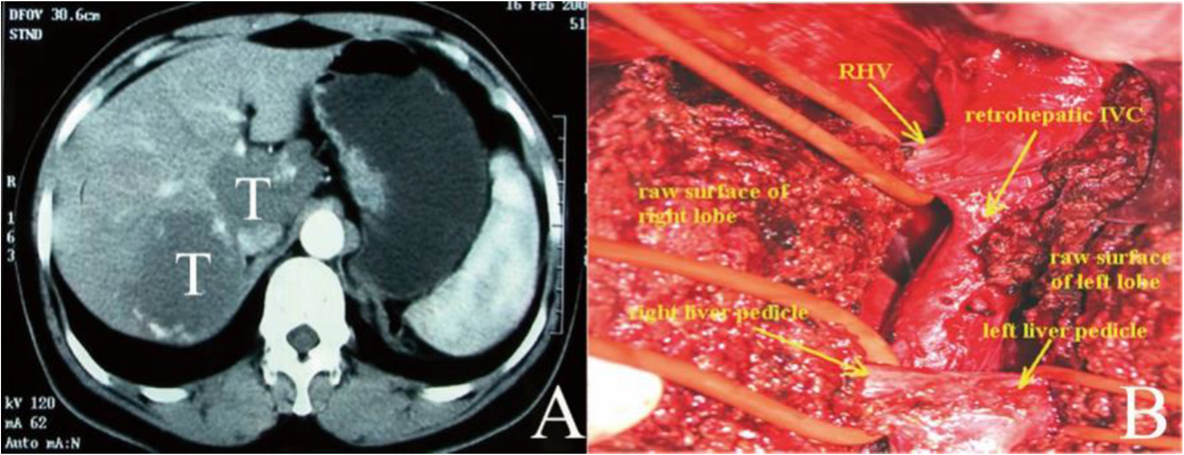
Complete resection of caudate lobe combine with S6 and S7 through anterior transhepatic approach (CT scan showed hemangioma in the whole caudate lobe and S6, 7. The intraoperative picture showed all the hemangioma removed)

There are 30 patients in 3 cm < D ≤ 6 cm group received operation of which approaches are mainly isolated complete resection of caudate lobe (*n* = 14) and partial resection (*n* = 13). The other three cases that combined with hemangioma in right liver, S5 segment and S6 + S7 segments received combined right liver resection, S5 segment resection, and S6 + S7 segment resection, respectively (Fig. 2).

**Fig. 3:**
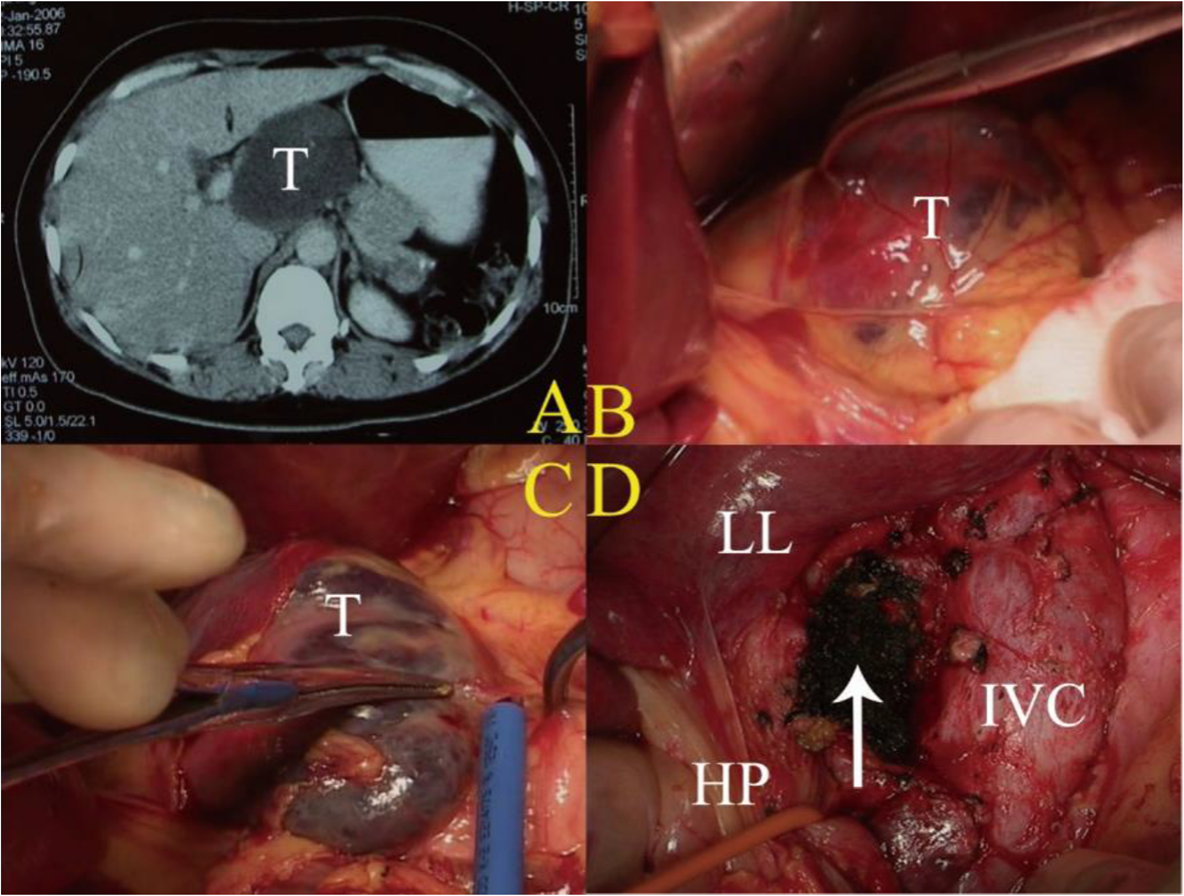
Isolated complete resection of caudate lobe (**a** CT scan showed hemangioma in the whole caudate lobe. **b** and **c** Showed the hemangioma intraoperatively, T = tumor. **d** The whole caudate lobe removed, HP = hepatic pedicle, LL = left liver, IVC = inferior vena cava)

There are 65 patients operated in 6 cm < D ≤ 9 cm group mainly by the approach of isolated complete caudate lobe resection (*n* = 51) (Fig. 3). Other approaches were also performed including combined left liver resection in 2 cases, combined right liver resection in 3 cases, and combined liver segment resection in 9 cases.

**Fig. 4:**
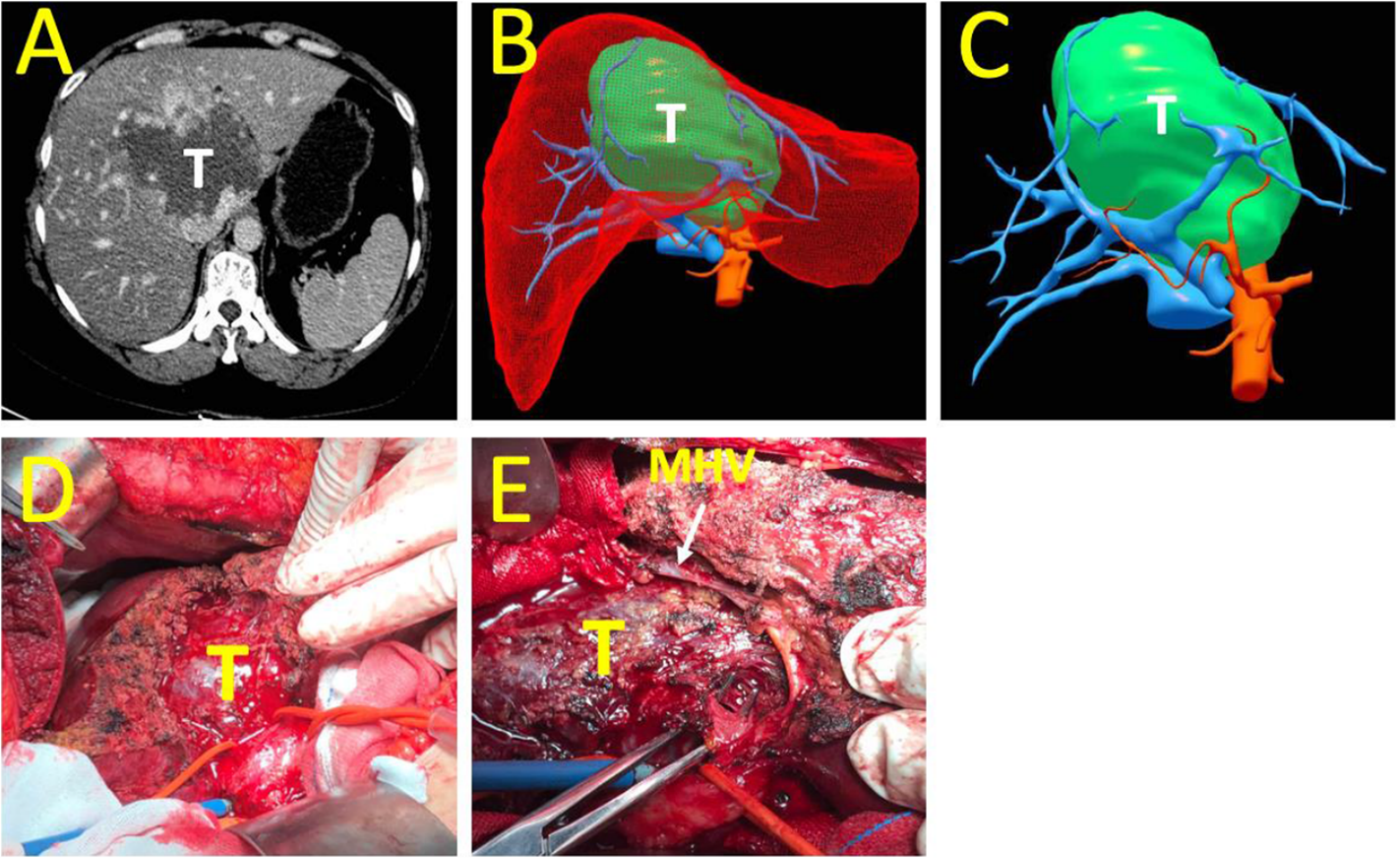
Isolated complete resection of caudate lobe (**a** CT scan showed a giant hemangioma in the whole caudate lobe. **b** and **c** Tumor vied by 3D reconstruction, T = tumor. **d** Tumor revealed through anterior transhepatic approach. **e** Tumor was beside the middle hepatic vein (MHV) and almost resected)

Eight patients whose tumors were larger than 9 cm in diameter received surgical treatment, including isolated complete resection in 5 cases, combined left liver resection in 1 case, and combined right liver resection in another (Fig. 4).

All patients received surgical treatment went through operation smoothly and successfully; the average operation time is 190 min (105–310 min), the average blood loss in operation is 490 ml (90–1100 ml), and the average hospital stay is 7 days (4–14 days). Postoperative complication including biliary leakage in 2 cases, intraperitoneal fluid in 5 cases, and pulmonary infection in 1 case were all cured by conservative treatment and no death occurred in patients operated (Table 3). No significant difference of incidence of postoperative complications is found among four groups of patients, partly because the number of cases with postoperative complications is too small.

**Table 3:** Intraoperative and postoperative outcomes

Surgical time (min) Median (range)	190 (105–310)
Blood loss (mL) Median (range)	490 (90–1100)
Postoperative hospital stay (day) Median (range)	7 (4–14)
No. of major complications	7.6 (8/105)
Postoperative hemorrhage	0
Bile leak	2
Intra-abdominal collection	5
Pleural effusion	1
Hospital mortality	0

### Follow-up and postoperative care

All cases were followed up periodically no less than 3 years in total and cases followed less than 6 months were recorded as lost to follow-up. Qualified follow-up files of 124 in 137 patients (90.5%) were acquired. Key variations in the follow-up were tumor growth, occurrence of clinical features etc. in patients with conservative treatment and relief of symptoms, and recurrence of hemangioma etc. in patients operated. Among patients in conservative treatment group, 7 cases turn to surgical treatment due to tumor growth and appearance of clinical symptoms and the other 32 cases had no obvious disorder within 3 years during follow-up. All patients got operation were clinical relieved.

## Discussion

Hepatic hemangioma is the most common benign hepatic tumor. In recent years, the common standard of clinical indication of treatment, risk assessment, and selection of therapies for hepatic hemangioma has been reached among researchers and surgeons [12]. However, clinical confusions exist on indication and approaches of treatment for hepatic hemangioma and mistreatment occurs even cause severe damage to patients’ in physical and mental interests sometimes [13, 14].

The caudate lobe is a special structure of liver, lying among major vascular structures, the IVC, the portal triads, and the hepatic venous confluence. Even though hepatic hemangioma accounts for a great proportion of diseases of the caudate lobe, the holistic incidence of hemangioma in the caudate lobe remains respectively small. Existing state of affairs makes it relatively more difficult to accumulate clinical experiences. With the anatomic traits of the caudate lobe and technique challenges of surgical procedures, standardization and normalization of the treatment for hemangioma in caudate lobe seem vaguer.

In this study, we analyzed 137 cases diagnosed with hepatic hemangioma in caudate lobe and treated in 5 medical centers during the past 25 years retrospectively for the first time, we analyzed and summarized the correlation between the clinical manifestation and the size of tumor of hepatic hemangioma in caudate lobe and based on the research, we propose new strategies of classification, surgical indication, and operation procedure.

### Clinical features of liver hemangioma in caudate lobe

Liver hemangioma is predominantly single in caudate. In this study, 86.1% cases were diagnosed with single liver hemangioma in caudate lobe. Tumors larger than 3 cm in diameter always occupy the entire caudate lobe and its growth pattern is mainly along the vena cava to the inside of liver (Figs. 1 and 4) or along the Spigelian lobe to the left side. When the tumor grows along and compresses the vena cava, symptoms including upper abdominal discomfort and dull pain appear. Tumors along the Spigelian lobe tend to exert pressure to small curvature of the stomach leading to epigastric distention, nausea, vomiting etc. Abdominal dull pain is also likely to present when capsule of caudate lobe is stimulated by the tumor.

### Classification of liver hemangioma in caudate lobe

The diameter and number of the tumor are the main basis for the clinical classification of liver hemangioma. Foreign recommendation suggests 4 cm in diameter as the demarcation point, while the domestic guideline recommends 5 cm in diameter [15–17]. Based on diameter of the tumor, it is recommended to classify liver hemangioma to three grades: small hemangiomas (D ≤ 5 cm), large hemangiomas (5 cm<D ≤ 10 cm), and giant hemangiomas (D> 10 cm) by Chinese experts [17]. And when analyzing the correlation between tumor size and clinical symptoms, there are few clinical manifestations of small liver hemangiomas.

However, due to the special nature of the location of hepatic caudate lobe, hemangiomas in this structure can stimulate liver capsule and compress adjacent part of small curvature of stomach or vena cava, causing symptoms related. This trait makes significant difference between liver hemangiomas in caudate lobe different from other liver hemangiomas. According to our analysis in this study, we realized that symptoms of liver hemangioma in caudate lobe are closely related to its size. The incidence of symptoms is 6.25% (1/16), 60.9% (28/46), 89.6% (60/67), and 100% (8/8) in patients with caudate lobe hemangioma in groups of D ≤ 3 cm, 3 cm<D ≤ 6 cm, 6 cm < D ≤ 9 cm, and D > 9 cm, respectively. Therefore, we believe that the current classification standard is not suitable for liver hemangioma in caudate lobe.

We recommend a new clinical classification of which divides liver hemangiomas in caudate lobe into four types (Table 4): type I, isolated hepatic hemangioma in caudate lobe, divided into four grades: type Ia, D ≤ 3 cm, type Ib, 3 cm < D ≤ 6 cm, type Ic, 6 cm < D ≤ 9 cm, and type Id, D > 9 cm; type II, hepatic hemangioma in caudate lobe combined with hepatic hemangioma in other segments; and type III, hepatic hemangiomas in caudate lobe combined with hepatic hemangioma in left (IIIa) or right liver (IIIb).

**Table 4:** Classification of hemangioma in the caudate lobe

Type		Location	Size
I	Ia	Caudate lobe	D ≤ 3 cm
	Ib	Caudate lobe	3 cm < D ≤ 6 cm
	Ic	Caudate lobe	6 cm < D ≤ 9 cm
	Id	Caudate lobe	D > 9 cm
II		Caudate lobe and other segment	
III	IIIa	Caudate lobe and left liver	
	IIIb	Caudate lobe and right liver	

### Treatment for liver hemangioma in caudate lobe

At present, there is no standard to help decide whether it is appropriate to apply surgical treatment for liver hemangioma in caudate lobe. The view in the past that large hepatic hemangioma larger than 10 cm and hepatic hemangioma larger than 5 cm at the edge of the liver require surgery has been gradually denied in recent years [18].

Especially in a most powerful article recently, Schnelldorfer T [19] conducted a follow-up study of 492 patients with hepatic hemangioma larger than 4 cm in diameter at the Mayo Clinic for at least 10 years and found that during the observation period, 2% of patients in the conservative group were threatened by deadly complications during the observation period and another 20% of patients present with clinical symptoms, while the group underwent surgery had a perioperative complication incidence of only 14%, but 7% of patients had life-threatening complications. Therefore, the authors believed that applying surgical procedure for liver hemangioma should be considered cautiously.

Affected by foreign opinions, the current mainstream opinion in China is that regardless of tumor size, conservative therapy and regular follow-up instead of surgical procedure are recommended for asymptomatic patients. Indications of surgery are [18, 20–22] as follows: (1) liver hemangioma of any size combined with spontaneous rupture or other accidents, presenting certain hepatic subcapsular hemorrhage or abdominal hemorrhage with hemorrhagic shock; (2) clinical symptoms such as abdominal pain and abdominal mass or consumptive coagulopathy (hemangioma-thrombocytopenia syndrome) combined with caudate lobe hemangioma; (3) women preparing for pregnancy with subcapsular large hemangiomas, to avoid the risk of tumor rupture and hemorrhage during pregnancy, preventive surgical resection is recommended; (4) emergency situation that needs urgent life-saving treatment; (5) lesions that cannot be distinguished between benign or malignant disease, especially when combined with type B hepatitis or cirrhosis; and (6) for asymptomatic patients who strongly require surgery, surgery is not recommended.

However, there had been no guidelines for the treatment of hemangioma in the caudate lobe. With reference to the experience and procedures of treatment for hepatic hemangioma, we analyzed the correlation between tumor size and clinical symptoms of 137 cases with caudate lobe hemangioma in this study. Our discussion of the treatment strategies for liver hemangioma in caudate lobe are as followed:

1) Conservative treatment is recommended for patients with tumor no bigger than 3 cm in diameter (type Ia).

2) For tumors of type Ib (3 cm < D ≤ 6 cm), we suggest that asymptomatic patients including those who strongly require surgery for psychological reasons should receive conservative treatment and follow-up, while patients with clinical symptoms which are usually mainly caused by tumor compression in this group are suitable for surgical approaches such as partial or isolated complete resection of caudate lobe.

3) For tumors of type Ic (6 cm < D ≤ 9 cm), surgery should be considered firstly in both symptomatic and asymptomatic patients. These patients basically have different clinical symptoms. In this case, the surgical approach is mainly isolated complete resection of caudate lobe.

4) For patients with tumors of type Id (D > 9 cm), it is recommended to choose surgery. In this study, all patients with tumors of type Id appeared with clinical symptoms and were in worse condition compared with other groups.

5) Liver hemangioma in caudate lobe combined with and other hepatic hemangioma (type II, type III) is also not rare and for patients in this situation, surgical therapy requires much more carefulness. First, it is necessary to judge whether the indications for surgery are met. The second is to figure out the causes of symptoms, to find out whether the hemangioma in caudate lobe or the rest part of the liver induced clinical symptoms. The third step is to determine whether it is appropriate to perform united resection combining the caudate lobe and other parts of the liver.

In this study, 19 cases were classified into type II and type III and of these patients, the smallest hemangioma in the rest of the liver is also bigger than 6 cm in diameter, some other hemangiomas even occupy half of the liver. Therefore, for patients with tumors of type II or type III, we performed liver resection of caudate lobe combined with other liver segments, the left or the right liver. However, the number of cases is small in our study; further accumulation is needed for more specific guidance for this situation.

In recent years, interventional therapy (transhepatic arterial embolization, TAE) increased in the treatment of hepatic hemangioma. It is reported [23] that TAE can effectively occlude small branches of the blood supply artery and lead to hemangioma fibrosis, in order to stop tumor growth and promote tumor shrinkage; it can alleviate clinical symptoms and achieve therapeutic goals. We have not adopted this technique yet, but interventional therapy is still recommended considering the difficulty of isolated complete caudate lobe resection. Radiofrequency ablation (RFA) [24] shows certain value in the treatment of hepatic hemangioma. It has been reported that RFA successfully treated giant hepatic hemangioma bigger than 10 cm in diameter. However, due to the special anatomical location of the caudate lobe, the implementation of RFA in liver hemangioma in caudate lobe is very difficult. We have not applied this technique for caudate lobe hemangioma yet.

### Surgical strategies for liver hemangioma in caudate lobe

With cutting-edge surgical instruments and developing techniques, caudate lobe is no longer an exclusive zone for surgery. However, how to perform surgeries on caudate lobe effectively and safely and reduce complications related needs further improvement. According to the experience of our surgical team, as for approaches, we highly recommend bilateral approach as a relatively safer way compared with left or right single-lateral procedure and anterior transhepatic approach splitting the liver through the middle liver fissure is not recommended unless it necessary for it is accompanied with more risks and requires more proficient skills. Former studies suggested that hemorrhage and blood transfusion during the operation are risk factors for postoperative complications of hepatic hemangioma [25].

Therefore, we believe that there are two key points for surgery treating liver hemangioma in caudate lobe: the first one is doing well in blood flow control to prevent bleeding; the second one is to streamline the surgical approach to reduce bleeding and liver damage. Since the caudate lobe resection was performed in 1994 by our team, we have gradually improved the blood flow control techniques in surgeries of caudate lobe, including preplacing tapes around first and second porta hepatis, intrahepatic IVC control, and establishing post-hepatic tunnel, which are gradually becoming routine preventive measures for difficult hepatectomy [1, 5].

So how can we actually streamline the surgical approach of caudate resection and reduce bleeding and unnecessary liver dissociation and damage in surgery? Hemorrhage mainly happens in two steps during caudate resection; one is when dealing with hepatic short veins in third hepatis and the other is when removing the caudate lobe from its junction with the liver. Reducing bleeding is the key point in these two phases; therefore, we tried to improve the traditional left, right, and left/right combined approaches, guiding the operative strategy in the way of basing on the essence of surgery manner [7, 9].

That is to separate caudate lobe from the four surrounding surfaces which are triple portal system in first porta hepatis, division of the top of caudate lobe and middle and left liver vein in second porta hepatis, short hepatic vein system in third porta hepatis, and conjunctions between caudate lobe and left and right liver, of which the treatment of the short venous system is the key step. In addition, when separating the caudate lobe and the left and right hepatic junctions, the hemorrhage is minimal when cutting along the Peng’s line or tip-to-process [7, 9], for liver tissues are thin and horizontal in this structure and the section is the smallest.

With the development of new minimal invasive technical means like transcatheter arterial embolization (TAE) and radiofrequency ablation (RFA), more effective options are available during treatment for patients with caudate lobe diseases. At the present, our department still takes surgical operations as the main method treating caudate lobe diseases and we are looking for further research combining interventional therapies with surgical procedure or using interventional techniques to even replace surgery in some patients.

## Conclusion

Hepatic hemangioma is the most common benign tumor in the liver, but has not been well studied especially for the hemangioma in the caudate lobe. Because of its special location and low coincidence, no specific standard and little thorough discussion of the treatment for liver hemangioma in caudate lobe have been reported. By retrospective analysis of the 137 cases, we intend to offer a reasonable reference for the standardization and normalization of the treatment for liver hemangioma in caudate lobe. As the research sample is small, more accumulation of cases and perspective studies are required to improve our study and understand of the disease.

## Data Availability

The data and materials were available to email the author.
